# Acceptability of integration of cervical cancer screening into routine HIV care, associated factors and perceptions among HIV-infected women: a mixed methods study at Mbarara Regional Referral Hospital, Uganda

**DOI:** 10.1186/s12913-023-09326-6

**Published:** 2023-04-03

**Authors:** Mackline Ninsiima, Agnes Nyabigambo, Joseph Kagaayi

**Affiliations:** 1 Department of Epidemiology and Biostatistics, School of Public Health, Makerere University, Kampala, Uganda; 2 Department of Community Health and Behavioural Sciences, School of Public Health, Makerere University, Kampala, Uganda; 3 Rakai Health Sciences Program, Kalisizo, Uganda

**Keywords:** Acceptability, Integration, Cervical Cancer Screening, HIV

## Abstract

**Background:**

Integrating cervical cancer screening into routine Human Immunodeficiency Virus (HIV) care has been endorsed as an effective strategy for increasing uptake of cervical cancer screening, facilitating early detection and treatment of pre-cancerous lesions among HIV-infected women. In Uganda, this strategy has not been implemented yet in most HIV clinics. Assessing acceptability of this intervention among HIV-infected women is of great relevance to inform implementation. We assessed acceptability of integration of cervical cancer screening into routine HIV care, associated factors and perceptions among HIV-infected women enrolled in the HIV clinic at Mbarara Regional Referral Hospital.

**Methodology:**

A mixed methods study utilizing explanatory sequential approach was conducted among 327 eligible HIV-infected women. Acceptability of integration of cervical cancer screening into routine HIV care was measured based on Theoretical Framework of Acceptability. Quantitative data was collected using a pre-tested questionnaire. We conducted focus group discussions to explore perceptions regarding the intervention among purposively selected HIV-infected women. Modified Poisson regression with robust variance analysis was utilized to determine factors associated with acceptability of the intervention. Statistical significance was determined at *p*-value <0.05. Thematic analysis utilizing inductive coding was applied to analyse qualitative data.

**Results:**

The majority of HIV-infected women (64.5%) accepted integration of cervical cancer screening into routine HIV care. Religion, perceived risk of developing cervical cancer and ever screened for cervical cancer were statistically significantly associated with acceptability of integration of cervical cancer screening into routine HIV care. Perceived benefits of the proposed intervention were: convenience to seek for cervical cancer screening, motivation to undergo cervical cancer screening, improved archiving of cervical cancer screening results, confidentiality of HIV patient information, and preference to interact with HIV clinic health workers. Shame to expose their privacy to HIV clinic health workers and increased waiting time were the only perceived challenges of the integrated strategy.

**Conclusion:**

Study findings highlight the need to take advantage of this acceptability to prioritize implementation of integration of cervical cancer screening into routine HIV care. HIV-infected women should be reassured of confidentiality and reduced waiting time to increase uptake of integrated cervical cancer screening and HIV services among HIV-infected women along the continuum of HIV care and treatment services.

## Background

Globally, cervical cancer is the fourth most frequently diagnosed cancer and fourth leading cause of cancer death in women with an estimated 570,000 incident cases and 311,000 deaths [1]. World Health Organisation (WHO) reported a high mortality rate from cervical cancer globally at an age-standardized rate of 6.9/100,000 [2]. In low and medium Human Development Index regions, cervical cancer ranks second to breast cancer among females at world age-standardized incidence and mortality rates of 18.2/100,000 and 12.0/100,000 respectively [1]. In Africa, cervical cancer is the most common cause of cancer accounting for 22% of all female cancers [3]. Of note, 34 out of every 100,000 women are diagnosed with cervical cancer, and 23 out of every 100,000 women die from cervical cancer every year [3]. The cervical cancer incidence and mortality rates have remained high in Sub-Saharan Africa [1, 4]. In Eastern Africa, age-standardized incidence and mortality rates of cervical cancer are 40.1/100,000 and 30.0/100,000 respectively [1]. In Uganda, prevalence of human papillomavirus among women is 33.6% with one of the highest cervical cancer incidence rates in the world of 47.5 per 100,000 per year [5].

Human Immunodeficiency Virus (HIV) has been associated with high vulnerability of developing cervical cancer, a great public health challenge. Since 1989, research studies have reported an increased incidence of cervical cancer among HIV-infected women [6]. HIV-infected women are at increased risk of new and persistent human papillomavirus infections hence an accelerated advancement and incidence of cervical cancer compared to HIV uninfected women [2, 7–12]. HIV-infected women develop cervical cancer 5 to 10 years earlier compared to HIV uninfected women [2]. Susceptibility to human papillomavirus infection and progression to cervical cancer is highly attributed to weakened cellular immunity among HIV-infected women [2, 6, 8]. Due to the association between HIV and cervical cancer, WHO recommended that all sexually active girls and women should undergo cervical cancer screening as soon as they are diagnosed HIV positive and further emphasized regular screening and treatment of pre-cancer lesions among HIV infected women [13]. With alignment to WHO guidelines, cervical cancer screening using Visual Inspection with Acetic acid and treatment of pre-cancerous cervical lesions by cryotherapy was recommended for all HIV-infected sexually active girls and women at enrolment into HIV care and repeated annually in Uganda [14]. Nevertheless, reports have indicated persistent low uptake of cervical cancer screening among this highly susceptible population [15, 16]. A nationally representative population-based survey carried out in Uganda reported that only 30.3% HIV-infected women had received cervical cancer screening [17].

Integration of cervical cancer screening into routine HIV care has been endorsed as an effective strategy to increase uptake of cervical cancer screening, achieve early detection and treatment of pre-cancerous lesions among HIV-infected women in Sub Saharan Africa where cervical cancer burden parallels that of HIV [4, 15–22]. In Uganda, this strategy has not been implemented yet in most HIV clinics. It has been noted that published studies from Uganda explored perceptions and preferences of health care providers, policy makers, and community members including women, men, and village health teams, regarding integration of HIV and cervical cancer screening services in a single-visit approach without focussing on HIV-infected women [23, 24]. Utilizing these findings might be difficult to understand perceptions of HIV-infected women enrolled in HIV care which would be instrumental in guiding implementation phase. Furthermore, acceptability of this intervention has not been assessed among HIV-infected women using Theoretical Framework of Acceptability (TFA); the approved framework for assessing acceptability of healthcare interventions. This indicates the need to generate scientific evidence regarding acceptability of the intervention among targeted recipients. Assessing acceptability of this proposed intervention is of great relevance to inform implementation into comprehensive HIV programming [25].

HIV-infected women enrolled in the HIV clinic are either referred or voluntarily undergo cervical cancer screening conducted by health workers in the cervical cancer screening department at Mbarara Regional Referral Hospital. Despite establishing referral or client-initiated cervical cancer screening system at Mbarara Regional Referral Hospital for the last 10 years, only 11.3% of HIV-infected women enrolled in the HIV clinic at Mbarara Regional Referral Hospital had undergone cervical cancer screening by September 30, 2019. HIV-infected women miss cervical cancer screening opportunities for early detection of pre-cancerous cervical lesions at enrolment and during subsequent annual intervals despite their frequent visits to HIV clinics for medical reviews, viral load monitoring, and monthly Anti–Retroviral Treatment (ART) refills. Such missed opportunities increase risk of presenting late with advanced cervical cancer and poor prognosis among this vulnerable population. Implementation of integration of cervical cancer screening into routine HIV care is expected to increase uptake of cervical cancer screening services at enrolment and during subsequent annual HIV clinic visits among HIV-infected women enrolled in the HIV clinic at Mbarara Regional Referral Hospital compared to currently practiced referral or client-initiated cervical cancer screening system. We assessed acceptability of integration of cervical cancer screening into routine HIV care, associated factors and perceptions among HIV-infected women enrolled in the HIV clinic at Mbarara Regional Referral Hospital.

## Methods

### Study site

The study was conducted in the HIV clinic at Mbarara Regional Referral Hospital in Mbarara District, South Western Uganda. Cervical cancer is the leading gynaecological cancer at Mbarara Regional Referral Hospital [26]. With a proportion of 25.2%, cervical cancer is the single leading cancer; contributing to 10.1% of all diseases on the gynaecological ward and 73.9% of all gynaecological cancers at Mbarara Regional Referral Hospital [26]. The HIV clinic in Mbarara Regional Referral Hospital has provided HIV care since November 1998. The clinic has two sections: adult care section, under the Department of Internal Medicine and paediatric and adolescents care section under the Department of Paediatrics. A total of 7,212 HIV-infected women were reported to be active in HIV care by September 30, 2019. On average 168 HIV-infected women receive HIV care and treatment services on a typical clinic day. HIV-infected women enrolled in the HIV clinic are either referred or voluntarily undergo cervical cancer screening conducted by health workers in the cervical cancer screening department at Mbarara Regional Referral Hospital. It is on these grounds that the HIV clinic at Mbarara Regional Referral Hospital was selected to study acceptability of integration of cervical cancer screening into routine HIV care, associated factors and perceptions among HIV-infected women.

### Study design

Mixed methods study design utilizing explanatory sequential approach was used to assess acceptability of integration of cervical cancer screening into routine HIV care, associated factors and perceptions among HIV-infected women. Using explanatory sequential approach, the quantitative phase of the study was first conducted followed by analysis of data. HIV-infected women were then selected based on generated quantitative results of acceptability of integration of cervical cancer screening into routine HIV care; to participate in qualitative investigations.

### Study population

This study was conducted among HIV-infected women receiving HIV care and treatment services from the HIV clinic at Mbarara Regional Referral Hospital. Only HIV-infected women aged 18 and above who turned up for ART refills at the HIV clinic on interview dates were included in the study. HIV-infected women aged 18 and above who turned up for ART refills on unscheduled dates were excluded based on electronic appointment lists generated for the respective appointment dates. 

### Rationale

HIV-infected women who turned up on unscheduled dates were excluded because they were not on the appointment list from which study respondents were systematically selected to participate in the study.

### Quantitative phase

#### Measurement of study variables

##### Dependent variable

The dependent variable for this study was acceptability of integration of cervical cancer screening into routine HIV care. The Theoretical Framework of Acceptability was adapted to measure acceptability of integration of cervical cancer screening into routine HIV care. The Theoretical Framework of Acceptability was proposed as a multi-component framework and systematic approach to assess intervention acceptability across prospective, concurrent, and retrospective temporal perspectives [25]. Questions based on Theoretical Framework of Acceptability constructs namely: affective attitude, burden, perceived effectiveness, ethicality, intervention coherence, opportunity costs, and self-efficacy were administered to eligible participants. Responses were based on a 5-point rating ordinal scale per construct. The summated score of constructs of acceptability per study participant was computed by summing weights assigned to construct responses; henceforth a continuous dependent variable.

##### Independent variables

Information on sociodemographic characteristics, awareness of cervical cancer, knowledge of risk factors of cervical cancer, knowledge of symptoms of cervical cancer, cervical cancer screening awareness, uptake of cervical cancer screening, and perceived risk of cervical cancer was obtained. Sociodemographic characteristics included age, marital status, highest education level, religion, area of residence, number of living biological children, and duration of HIV disease.

To assess knowledge of risk factors and symptoms of cervical cancer, participants were requested to select from response options “1. Yes” “2. No” for each item. A correct answer was awarded “1” whereas a wrong answer was awarded “0”. According to the African Women Awareness of CANcer tool, codes to response options were assigned without any meaning. So, out of two options, if the participant selected an appropriate answer, one received an award of "1" for the item because it was the correct answer. If the participant selected an inappropriate answer, one received an award of "0" for the item because it was the wrong answer. A summated score was obtained for variables: knowledge of risk factors and knowledge of signs and symptoms of cervical cancer.

Knowledge of risk factors of cervical cancer was categorised into two categories: “1. Poor Knowledge” and “2. Good Knowledge”; based on the mean score as evidenced in recent published studies [27, 28]. “1. Poor Knowledge” was awarded to HIV-infected women whose summated score of weights of items of knowledge of risk factors of cervical cancer was less than the mean score value of 8.34. “2. Good Knowledge” was awarded to HIV-infected women whose summated score of weights of items of knowledge of risk factors of cervical cancer was greater than or equal to the mean score value of 8.34.

Knowledge of signs and symptoms of cervical cancer was also categorised into two categories: “1. Poor Knowledge” and “2. Good Knowledge”; based on the mean score as demonstrated in recent published studies [27, 28]. “1. Poor Knowledge” was awarded to HIV-infected women whose summated score of weights of items of knowledge of signs and symptoms of cervical cancer was less than the mean score value of 7.99. “2. Good Knowledge” was awarded to HIV-infected women whose summated score of weights of items of knowledge of signs and symptoms of cervical cancer was greater than or equal to the mean score value of 7.99.

#### Sample size and sampling

For the quantitative component, sample size was calculated using sample size estimation formula [29, 30]. The sample size was determined by assuming a 2-sided type 1 error rate of 5%. The calculated standard deviation in cervical cancer screening was 8.75 based on sample size estimation for epidemiological studies [30]. Assuming a marginal error of less than one, the minimum number of participants required was 294. After considering a 10% non-response rate, the resulting sample size was 327 participants. Systematic sampling method was used to select participants among HIV-infected women who had been scheduled to turn up at the HIV clinic for ART refills based on respective appointment dates.

#### Data collection

Quantitative data were collected using administered questionnaires. The pre-coded questionnaire was pre-tested at Kawaala Health Centre IV to check for suitability of various aspects such as translations, skip procedures, filtering questions and modifications were made thereafter. The questionnaire was translated into Runyankole/Rukiiga. Selection and recruitment of experienced research assistants was based on competence, quantitative data collection skills and qualifications. Research assistants received training on how to administer study questions before data collection. After meeting selected participants to take part in the study, research assistants introduced themselves and explained the purpose of the study. Eligible participants provided written informed consent before the interview was conducted. Each questionnaire was completed within 15 - 20 minutes. During data collection, questionnaires were reviewed on completion of each interview so that corrections are made in addition to checking for completeness before departure of participants. Supervision was conducted to ensure compliance throughout the study.

#### Data management

Data capture screens with in-built checks for consistency, logical flow, range and accuracy of data were designed in EpiData version 3.0 for data entry. Data were cleaned and stored daily. Double-entry of data was done to check for any errors. Stored data was backed up on different flash discs. Questionnaires were kept securely throughout the study and thereafter.

#### Data analysis

Linear regression analysis was the recommended method of modelling since the dependent variable, acceptability of integration of cervical cancer screening into routine HIV care, was a continuous variable. Summated scores of acceptability of integration of cervical cancer screening into routine HIV care were converted into percentages. The regression modelling predicting acceptability of integration of cervical cancer screening into routine HIV care from all independent variables was conducted. Residuals were generated followed by testing assumptions of linear regression: normality of residuals and homogeneity of variance. Linear regression analysis was not utilised for this study because both assumptions of normality and homogeneity of variance of residuals were violated.

Acceptability of integration of cervical cancer screening into routine HIV care was dichotomized into two categories: “0. Not Accepted” and “1. Accepted” based on 26.25, the 75^th^ percentile of the highest possible summated score of 35 from seven constructs of Theoretical Framework of Acceptability. The 75^th^ percentile was recommended as the cut-off value based on a published study which utilised likert-type scale responses assessing constructs to measure composite dependent variable [31]. “0. Not Accepted” was awarded to HIV-infected women whose summated score of weights of constructs of acceptability of integration of cervical cancer screening into routine HIV care was less than 26.25. “1. Accepted” was awarded to HIV-infected women whose summated score of weights of constructs of acceptability of integration of cervical cancer screening into routine HIV care was greater than or equal to 26.25. Frequencies and percentages of HIV-infected women who had accepted and those who had not accepted integration of cervical cancer screening into routine HIV care were computed.

#### Univariable analysis

All variables were analysed to describe study respondents. For independent categorical variables, data were presented as frequencies and percentages.

#### Bivariable analysis

Cross tabulations of outcome variable and independent variables were done to obtain frequencies and corresponding percentages of HIV-infected women who accepted and those who did not accept the integration of cervical cancer screening into routine HIV care per independent variable. Selection of regression model to use at bivariable analysis was conducted. Using occupation as an example, logistic regression, log binomial and Modified Poisson regression models were applied. Logistic regression and log binomial models overestimated measures of association compared to Modified Poisson regression model. Given that the outcome had a higher percentage of 64.5%, Modified Poisson regression with robust variance was selected for bivariable analysis to obtain crude prevalence ratios, 95% confidence interval, and corresponding *p*-values.

#### Multivariable analysis

Multicollinearity among independent variables was checked for using variance inflation factor. Regression modelling predicting acceptability of integration of cervical cancer screening into routine HIV care from all independent variables was conducted and then vif. command applied to check for multicollinearity. Variance inflation factors for all independent variables were less than 2.5. Ultimately, all independent variables were included in multivariable analysis. Modified Poisson regression with robust variance using stepwise logical model building technique was conducted to obtain adjusted prevalence ratios, 95% confidence interval and corresponding *p*-values. Checking for goodness of fit of the model was done based on Akaike Information Criteria (AIC). Significance of independent variables was set at *p-value <*0.05. Statistical analysis was conducted using Stata/SE Version 14.0.

### Qualitative phase

#### Sample size and sampling

With reference to the 5–point likert-type scale, acceptability of integration of cervical cancer screening into routine HIV care was categorised into five categories: “1. Very Unacceptable”, “2. Unacceptable”, “3. Neutral”, “4. Acceptable” and “5. Very Acceptable” based on summated score of weights of constructs of acceptability. “1. Very Unacceptable” was awarded to respondents whose summated score of weights of constructs of acceptability was 7 and below. “2. Unacceptable” was awarded to respondents whose overall score of weights of constructs of acceptability was 8-14. “3. Neutral” was awarded to respondents whose summated score of weights of constructs of acceptability was 15-21. “4. Acceptable” was awarded to respondents whose overall score of weights of constructs of acceptability was 22-28. “5. Very Acceptable” was awarded to respondents whose summated score of weights of constructs of acceptability was 29 and above out of the 35 criteria.

Based on the category of acceptability of integration of cervical cancer screening into routine HIV care, 6 focus group discussions were conducted among HIV-infected women. The number of focus group discussions per level of acceptability of integration of cervical cancer screening into routine HIV care was based on number of HIV-infected women within respective categories. Sample size of 6 focus group discussions was further determined by data saturation point; a point at which further sampling did not generate any new concepts or ideas about the phenomenon under investigation. Purposive sampling was used to select HIV-infected women from the quantitative phase to participate in focus group discussions.

#### Data collection

The focus group discussion guide was specifically developed for this study with questions aimed at eliciting HIV-infected womens’ perceptions of integration of cervical cancer screening into routine HIV care. The focus group discussion guide was pre-tested and recommended changes were made to ensure that the guide captured relevant and appropriate information before use. Focus group discussion guide was translated into Runyankole/Rukiiga, the commonly used local language among study respondents. Key questions in the focus group discussion guide explored participants’ impressions, anticipated benefits, and challenges of integration of cervical cancer screening into routine HIV care in a single visit approach in HIV clinics in Uganda. An experienced research assistant at collecting qualitative data through conducting focus group discussion was recruited.

#### Data management

Focus group discussions were audio-recorded. The collected audio-recorded data were adequately and appropriately backed up. All audio recorded local language interviews were translated into English and transcribed verbatim simultaneously. The transcripts were proof-read before importing them into Atlas.ti Version 6.0, a qualitative data management software.

#### Data analysis

Thematic analysis method using inductive coding was used to analyse qualitative data. Exploration of data and synthesis of codes, subthemes and themes was done in Atlas.ti Version 6.0. Relevant verbatim quotations were selected as evidence to support generated themes.

## Results

### Socio-demographic characteristics of study respondents

Table 1 shows characteristics of study respondents. The majority of respondents (33.0%) were aged 40 – 49. Based on self-reports, 59.0% of respondents had lived with HIV for 10 years and above since they were diagnosed HIV positive. Of note, 75.8% reported that they were aware about cervical cancer screening programme whereas 65.1% had undergone cervical cancer screening.

**Table 1:** Characteristics of study respondents

**Study variables**	**Frequencies (*n*=327)**	**Percentages (%)**
**Completed age**		
18-29	43	13.2
30-39	91	27.8
40-49	108	33.0
50 and above	85	26.0
**Residence**		
Rural	197	60.2
Urban	130	39.8
**Highest education level**		
None	70	21.4
Primary Education	147	45.0
Secondary or Higher Education	110	33.6
**Marital Status**		
Not Married	205	62.7
Married	122	37.3
**Religion**		
Anglican	153	46.8
Catholic	107	32.7
Muslim	34	10.4
Others (Pentecostal & SDA)	33	10.1
**Occupation**		
Not working	28	8.6
Employed (Paid)	29	8.9
Self Employed (Businesswoman)	115	35.1
Self Employed (Agriculture)	155	47.4
**Number of children**		
None	26	8.0
1 – 3	183	56.0
4 and above	118	36.0
**HIV Duration (Number of years since diagnosed HIV positive)**		
1 – 4	50	15.3
5 – 9	84	25.7
10 and above	193	59.0
**Awareness of cervical cancer**		
Yes	311	95.1
No	16	4.9
**Knowledge of risk factors of cervical cancer**		
Poor Knowledge	149	45.6
Good Knowledge	178	54.4
**Knowledge of signs and symptoms of cervical cancer**		
Poor Knowledge	119	36.4
Good Knowledge	208	63.6
**Perceived risk of developing cervical cancer**		
Much Below Average	67	20.4
Below Average	28	8.6
Average	31	9.5
Above Average	31	9.5
Much Above Average	170	52.0
**Awareness of cervical cancer screening**		
Yes	248	75.8
No	79	24.2
**Ever screened for cervical cancer**		
Yes	213	65.1
No	114	34.9

### Acceptability of integration of cervical cancer screening into routine HIV care

The majority of HIV-infected women (64.5%) accepted integration of cervical cancer screening into routine HIV care. Of note, 35.5% did not accept integration of cervical cancer screening into routine HIV care.

#### Factors associated with acceptability of cervical cancer screening into routine HIV care

Table 2 shows adjusted prevalence ratios with corresponding confidence intervals. At multivariable analysis, based on 95% confidence intervals, religion, perceived risk of developing cervical cancer and ever screened for cervical cancer were statistically significantly associated with acceptability of integration of cervical cancer screening into routine HIV care among HIV-infected women at Mbarara Regional Referral Hospital. Muslims were 47% more likely to accept integration of cervical cancer screening into routine HIV care compared to Protestants. HIV-infected women with “Much Above Average” perceived risk of developing cervical cancer were 43% more likely to accept integration of cervical cancer screening into routine HIV care compared to HIV-infected women with “Much Below Average” perceived risk of developing cervical cancer. HIV-infected women who had not undergone cervical cancer screening were 29% less likely to accept integration of cervical cancer screening into routine HIV care compared to HIV-infected women who had undergone cervical cancer screening.

**Table 2:** Factors associated with acceptability of integration of cervical cancer screening into routine HIV care among HIV-infected women

**Independent variables**	**Acceptability of integration of cervical cancer screening into routine HIV care**		**Adjusted prevalence ratios**	**95% confidence intervals**
	**Not Accepted** **n (%)**	**Accepted** **n (%)**		
**Completed age**				
18-29	19 (44.2)	24 (55.8)	Ref.	
30-39	30 (33.0)	61 (67.0)	1.11	[0.83 – 1.49]
40-49	37 (34.3)	71 (65.7)	1.16	[0.86 – 1.56]
50 and above	30 (35.3)	55 (64.7)	1.15	[0.86 – 1.55]
**Residence**				
Rural	77 (39.1)	120 (60.9)	Ref.	
Urban	39 (30.0)	91 (70.0)	1.13	[0.97 – 1.31]
**Highest education level**				
None	28 (40.0)	42 (60.0)	Ref.	
Primary Education	58 (39.5)	89 (60.5)	0.88	[0.70 – 1.11]
Secondary or Higher Education	30 (27.3)	80 (72.7)	0.99	[0.78 – 1.26]
**Marital status**				
Not Married	75 (36.6)	130 (63.4)	Ref.	
Married	41 (33.6)	81 (66.4)	0.97	[0.82 – 1.15]
**Religion**				
Protestant	66 (43.1)	87 (56.9)	Ref.	
Catholic	34 (31.8)	73 (68.2)	1.16	[0.96 – 1.39]
Muslim	5 (14.7)	29 (85.3)	1.47	[1.21 – 1.78] ***
Others (Pentecostal & SDA)	11 (33.3)	22 (66.7)	1.14	[0.88 – 1.48]
**Occupation**				
None	7 (25.0)	21 (75.0)	Ref.	
Employed (Paid)	7 (24.1)	22 (75.9)	1.03	[0.80 – 1.33]
Self Employed (Business)	38 (33.0)	77 (67.0)	0.91	[0.73 – 1.14]
Self Employed (Agriculture)	64 (41.3)	91 (58.7)	0.81	[0.64 – 1.03]
**Number of children**				
None	13 (50.0)	13 (50.0)	Ref.	
1 – 3	50 (27.3)	133 (72.7)	1.25	[0.86 – 1.83]
4 and above	53 (44.9)	65 (55.1)	0.95	[0.63 – 1.43]
**HIV Duration (Number of years since diagnosed HIV positive)**				
1 – 4	24 (48.0)	26 (52.0)	Ref.	
5 – 9	26 (31.0)	58 (69.0)	1.18	[0.89 – 1.58]
10 and above	66 (34.2)	127 (65.8)	1.08	[0.81 – 1.44]
**Awareness of cervical cancer**				
Yes	104 (33.4)	207 (66.6)	Ref.	
No	12 (75.0)	4 (25.0)	0.46	[0.20 – 1.02]
**Knowledge of risk factors of cervical cancer**				
Poor Knowledge	61 (40.9)	88 (59.1)	Ref.	
Good Knowledge	55 (30.9)	123 (69.1)	1.01	[0.85 – 1.20]
**Knowledge of signs and symptoms of cervical cancer**				
Poor Knowledge	52 (43.7)	67 (56.3)	Ref.	
Good Knowledge	64 (30.8)	144 (69.2)	1.14	[0.96 – 1.35]
**Perceived risk of developing cervical cancer**				
Much Below Average	33 (49.3)	34 (50.7)	Ref.	
Below Average	10 (35.7)	18 (64.3)	1.27	[0.91 – 1.78]
Average	15 (48.4)	16 (51.6)	1.13	[0.75 – 1.70]
Above Average	11 (35.5)	20 (64.5)	1.23	[0.87 – 1.73]
Much Above Average	47 (27.6)	123 (72.4)	1.43	[1.11 – 1.85] **
**Awareness of cervical cancer screening**				
Yes	76 (30.7)	172 (69.3)	Ref.	
No	40 (50.6)	39 (49.4)	1.03	[0.74 – 1.45]
**Ever screened for cervical cancer**				
Yes	59 (27.7)	154 (72.3)	Ref.	
No	57 (50.0)	57 (50.0)	0.71	[0.58 – 0.86] **

^*^ *p<*0.05 ** *p<*0.01 *** *p<*0.001

### Perceptions of HIV infected women regarding integration of cervical cancer screening into routine HIV care

#### Characteristics of focus group discussion participants

Table 3 shows characteristics of Focus Group Discussion (FGD) participants. FGD participants were grouped into 6 groups based on level of acceptability to ensure homogeneity of groups. FGD 1 comprised of 2 and 4 participants from “Very Unacceptable” and “Unacceptable” levels of acceptability respectively. FGD 2 comprised of 6 participants from “Neutral” level of acceptability. FGD 3 comprised of 6 participants from “Acceptable” level of acceptability. FGD 4 comprised of 6 participants from “Acceptable” level of acceptability. FGD 5 comprised of 6 participants from “Very Acceptable” level of acceptability. FGD 6 comprised of 6 participants from “Very Acceptable” level of acceptability.

**Table 3:** Characteristics of focus group discussion participants

**Study variables**	**Frequencies (*n*=36)**	**Percentages (%)**
**Completed Age**		
Age [Mean Age (SD)]	40.0 (9.8)	
20-29	7	19.4
30-39	10	27.9
40-49	12	33.3
50 and above	7	19.4
**Residence**		
Rural	16	44.4
Urban	20	55.6
**Highest Education Level**		
None	6	16.7
Primary Education	17	47.2
Secondary or Higher Education	13	36.1
**Occupation**		
Not working	6	16.7
Employed (Paid)	6	16.7
Self Employed (Businesswoman)	11	30.5
Self Employed (Agriculture)	13	36.1
**Level of acceptability of integration of cervical cancer screening into routine HIV care**		
Very Unacceptable	2	5.6
Unacceptable	4	11.1
Neutral	6	16.7
Acceptable	12	33.3
Very Acceptable	12	33.3

Table 4 shows codes, sub-themes and themes of perceptions of HIV-infected women regarding integration of cervical cancer screening into routine HIV care. Perceptions of HIV-infected women were presented in two thematic areas: i) perceived benefits of integration of cervical cancer screening into routine HIV care and ii) perceived challenges of integration of cervical cancer screening into routine HIV care.

**Table 4:** Codes, sub-themes and themes

**Codes**	**Sub -Themes**	**Theme**
- Receiving HIV drugs and cervical cancer screening services on the same scheduled date - Receiving HIV care and cervical cancer screening services from the same place - Reduced disturbance and movements seeking for HIV care and cervical cancer screening services from different clinics	Convenience to seek cervical cancer screening services	Perceived benefits of integration of cervical cancer screening into routine HIV care
- Increased awareness about cervical cancer and the rationale to undergo cervical cancer screening services - Early detection and treatment of precancerous lesions - Compliance and adherence to undergo cervical cancer screening services annually - Increased opportunities to undergo cervical cancer screening services	Motivation to undergo cervical cancer screening	
- Record keeping of cervical cancer screening results and HIV related information in one HIV patient’s file	Improved archiving of cervical cancer screening results	
- Reduction in HIV stigma from the HIV uninfected women and non-HIV health workers at the cervical cancer screening unit - Privacy of the HIV positive status	Confidentiality of HIV patient information	
- Free interaction with HIV health workers whom the HIV-infected women are used to - Fear to disclose the HIV status to non-HIV health workers	Preference to interact with HIV clinic health workers	
- Ashamed to expose private parts to health workers who have known the HIV-infected women - Preference to open up and share experiences to health workers who don’t know them - Fear to continue interacting with the same health workers they have exposed their private parts for any other HIV related services	Shame to expose their privacy to HIV clinic health workers	Perceived challenges of integration of cervical cancer screening into routine HIV care
- Increase on time spent at the HIV clinic to undergo cervical cancer screening - Delay at the HIV clinic to receive both HIV and cervical cancer screening services	Increased waiting time	

### Perceived benefits of integration of cervical cancer screening into routine HIV care

#### Convenience to seek cervical cancer screening services

Convenience to seek cervical cancer screening services was the most predicted advantage of integration of cervical cancer screening into routine HIV care. In all focus group discussions, HIV-infected women acknowledged that integration of cervical cancer screening into routine HIV care would grant them an opportunity to receive both cervical cancer screening in addition to HIV care and treatment services from the HIV clinic compared to currently practiced referral or client – initiated cervical cancer screening conducted in the cervical cancer screening unit at Mbarara Regional Referral Hospital. HIV-infected women in the majority of focus group discussions revealed that referral or client – initiated cervical cancer screening services have been quite hectic and inconveniencing to an extent that some of them have missed several opportunities to undergo cervical cancer screening; hence supporting integrated mode of delivery of cervical cancer screening and HIV related services. Furthermore, HIV-infected women claimed that the proposed strategy of delivering cervical cancer screening would reduce disruption and movement from one clinic to another since all cervical cancer screening and HIV related services would be available and received under one roof in a single HIV clinic visit comprehensively.

“Sending you down there wouldn’t have a problem but it can somehow disturb you …, but if they are here, you can know that I am going to pick drugs and then test so I get all services from one place...” (Respondent 1, FGD 6 _ Very Acceptable)

HIV-infected women in most focus group discussions affirmed that convenience attributed to integration of cervical cancer screening into routine HIV care would eventually save time spent at the health facility compared to currently delivered stand-alone cervical cancer screening and HIV services.

“… because when she comes here, she has to pick drugs and then slope down there or first slope there and hurry back to get drugs and in this she may be caught by time, or even by the time she gets down, she may find that they have already closed but if those services are here, she can get drugs and later enter the room and they test for cancer of the cervix.” (Respondent 1, FGD 3 _ Acceptable)

HIV-infected women in most focus group discussions believe that scenarios of missing HIV care and treatment services due to disruption of seeking cervical cancer screening services from the cervical cancer screening unit at Mbarara Regional Referral Hospital would be minimised by implementation of integrated cervical cancer screening and HIV related services. It was further revealed that integrated services would save time since a client would easily receive both HIV and cervical cancer screening services in a single HIV clinic compared to spending a lot of time while travelling to the health facility twice; to seek for cervical cancer screening and HIV related services from two separate clinics on different days which would in due course reduce travel costs.

“Sending you down there wouldn’t have a problem but it can somehow disturb you because sometimes you can be home without money waiting to use the one you have the time you are coming to pick drugs, ... It can disturb you a bit if you go down there to get tested and then by the time you come back, you find the health workers here have closed. And you have to come back the next day to pick drugs… but if they are here, you can know that I am going to pick drugs and then test so I get all services from one place, I test for cancer of the cervix and at the same time, pick my drugs on the same day.” (Respondent 1, FGD 5 _ Very Acceptable)

#### Motivation to undergo cervical cancer screening

HIV-infected women in all focus group discussions reported that integration of cervical cancer screening into routine HIV care would motivate HIV-infected women to undergo cervical cancer screening services. This would ultimately increase uptake of cervical cancer screening among HIV-infected women; hence achieving the primary goal of implementing integration of cervical cancer screening into routine HIV care.

“When they bring the cervical cancer screening clinic here, it will be really so good for it will give us morale to access and undergo cervical cancer screening.” (Respondent 2, FGD 2 _ Neutral)

Furthermore, HIV-infected women in the majority of the focus group discussions alleged that integration of cervical cancer screening into routine HIV care would inspire HIV-infected women, targeted recipients to comply and adhere to recommended annual cervical cancer screening services. Since documentation about when one last conducted cervical cancer screening would be captured in the same file with all other HIV related information, it would be easier for health workers to remind clients about the appointment date for conducting the next cervical cancer screening procedure.

“… when you are coming to pick drugs, they also test you from here is really so good because I went down there and they tested me. They told me to go back … but like how you said it at the beginning, I have never gone back. But if it is here and I have come to pick drugs or I have come for treatment, they can check in my file and remind me that on such a date, you are supposed to come and they test you.” (Respondent 1, FGD 4 _ Acceptable)

#### Improved archiving of cervical cancer screening results

HIV-infected women in some focus group discussions acknowledged that integration of cervical cancer screening into routine HIV care would enhance improved record keeping of cervical cancer screening results. This was highly attributed to the fact that documentation about when the client last conducted cervical cancer screening would be captured in the same file with all other HIV related information.

“For me I see that the beauty about testing me from here is that everything will be done from here and all will be put in one file and anyone who gets your file will have to see all the diseases that you have.” (Respondent 5, FGD 5 _ Very Acceptable)

HIV-infected women in few focus group discussions anticipated that undergoing cervical cancer screening from the HIV clinic would make it quite easier for follow up of cervical cancer screening results.

“You see when we are examined from this our clinic, even when results don’t come there and then, …, when you come back, you know that you will find your results in your file and still the health worker will have the responsibility of telling you what the outcome of the results was, ….” (Respondent 3, FGD 5 _ Very Acceptable)

#### Confidentiality of HIV patient information

HIV-infected women in the majority of focus group discussions claimed that the proposed intervention would promote confidentiality of HIV status and any other health related information due to interaction with only health workers and fellow HIV-infected women; as opposed to the current situation where HIV-infected women are forced to interact with other health workers and HIV uninfected women at the cervical cancer screening unit of Mbarara Regional Referral Hospital. Confidentiality was very much appreciated as a great attribute of integrated delivery of cervical cancer screening comprehensively with all other routine HIV care services due to reduced stigma.

“You see when they are sending us down there; they send us with our files and remember there are people who have come so early to test for cancer of the cervix. …, they see people bombarding them and then they start to ask, where are these ones coming from? Ahh they are HIV positive women. You see them pointing fingers, ... But if it has come to our clinic and you know that if I have come to test for cancer of the cervix I will find services at the HIV clinic, I will also be confident wherever I will be seated because I will know that the people I am seated with are my fellows.” (Respondent 2, FGD 6 _ Very Acceptable)

#### Preference to interact with HIV clinic health workers

HIV-infected women in most focus group discussions appreciated integrated strategy whereby health workers from the HIV clinic will perform cervical cancer screening in addition to other HIV related services. HIV-infected women preferred receiving cervical cancer screening conducted by HIV clinic health workers to currently practiced system where cervical cancer screening procedure is conducted by health workers at the cervical cancer screening unit of Mbarara Regional Referral Hospital. HIV-infected women revealed that they can freely share their experiences or any health-related issues with health workers at the HIV clinic due to strong relationships built over time as opposed to health workers at the cervical cancer screening unit of Mbarara Regional Referral Hospital.

“..., me I think that if they bring them here, it will be easy for us …, not like down there because you find in most cases, when they would send us there, the health workers would look at you as if there is something on you, they would all be neglecting to attend to you, because the people down there may not be as free to you as this one who can be here, you can explain everything to her ...” (Respondent 3, FGD 3 _ Acceptable)

### Perceived challenges of integration of cervical cancer screening into routine HIV care

#### Shame to expose their privacy to HIV clinic health workers

HIV-infected women reported that they would prefer receiving cervical cancer screening conducted by health workers at the cervical cancer screening unit of Mbarara Regional Referral Hospital as opposed to health workers at the HIV clinic. They argued that due to having interacted for quite a very long time, they would always be overwhelmed with fear and shame to continue interacting with the same health workers to whom they have exposed their private parts for any other HIV related services. They confessed that they would honestly prefer health workers at the cervical cancer screening unit of Mbarara Regional Referral Hospital because they rarely interact with them; hence no fear and shame to expose their private parts.

“… ever since l started picking drugs from here, the nurse I found here is the one still there, the doctor I found there is the one still there, but there is a way we are created in our private parts, if someone has ever seen you, you feel ashamed as if she knows how you are exactly, as if she will tell others that so and so is like this and that, but down there where we go, sometimes you find they have changed them, you find that those who are always there are very different. And even by the time you go there, they can’t be remembering you because you go there once, but here, we come every month, every after two months, someone knows how you are created down, but down there, someone cannot even be remembering that you are the one but here they know us as patients in and out … you say that now that he has seen me, he has remembered how I look like down there in my private parts...” (Respondent 5, FGD 1 _ Very Unacceptable and Acceptable)

HIV-infected women anticipated that fear and shame to undergo cervical cancer screening conducted by health workers at the HIV clinic would even generate non-adherence to scheduled HIV clinic visits.

“Sometimes, those people will start irregular treatment. The day they gave her, if she knows that she is going to be tested for cancer of the cervix, she won’t come or that day when she is to come, she will come when she is in her periods.” (Respondent 4, FGD 2 _ Neutral)

#### Increased waiting time

HIV-infected women in the majority of focus group discussions argued that delivering cervical cancer screening comprehensively with other routine HIV care services during scheduled HIV clinic visits would be very time consuming hence increasing waiting time at the HIV clinic. With increased numbers of HIV-infected women enrolled into HIV care at the HIV clinic, participants were worried of potential delay at the HIV clinic attributed to integration of cervical cancer screening into routine HIV care.

“By the time you get through with the line of picking drugs, …, in fact you are already tired. And now imagine, you will also have to go the cancer room… don’t you think women will go back at night or even the next day [laughs] …” (Respondent 5, FGD 1 _ Very Unacceptable and Unacceptable)

## Discussion

This study assessed acceptability of integration of cervical cancer screening into routine HIV care, associated factors and perceptions among HIV-infected women enrolled in the HIV clinic at Mbarara Regional Referral Hospital. Analysis of data showed that 64.5% of HIV-infected women enrolled in the HIV clinic at Mbarara Regional Referral Hospital accepted integration of cervical cancer screening into routine HIV care. This study finding is lower than acceptance among HIV-infected women to undergo cervical cancer screening procedure reported at 87.6% after incorporating Visual Inspection with Acetic acid into the routine clinical services offered at Family AIDS Care and Education Services clinics in Kisumu, Kenya [32].

Acceptability of the proposed intervention at 64.5% is lower than cervical cancer screening acceptance of 79.8% among HIV-infected women at the HIV treatment centre, Nigerian Institute of Medical Research (NIMR), Lagos, Nigeria who participated in the study assessing acceptability of integration of cervical cancer screening into HIV care [8]. In addition, 64.5% is lower than 96.5% acceptability to undergo Visual Inspection with Acetic acid after integrating cervical cancer screening into HIV care and treatment services in a district hospital in Abuja, Nigeria [33]. This could be attributed to perceived challenges of integration of cervical cancer screening into routine HIV care among HIV-infected women enrolled at the HIV clinic.

Results of multivariable regression analysis indicated that religion, perceived risk of developing cervical cancer and ever screened for cervical cancer were statistically significantly associated with acceptability of integration of cervical cancer screening into routine HIV care among HIV-infected women. Despite the fact that Muslims accepted integration of cervical cancer screening into routine HIV care compared to Protestants, a study conducted among HIV-infected women reported that religion was not significantly associated with acceptability of integration of cervical cancer screening into routine HIV care [8]. Additionally, a study conducted in Ethiopia reported that religion differences were not significantly associated with acceptability and uptake of cervical cancer screening among HIV-infected women at St. Paul's and Zewditu Hospitals where cervical cancer screening had been integrated into HIV care and treatment services [34]. Regardless of the fact that this was a surprising finding, it is unclear why most Christians inclusive of Protestants consinder fatal illnesses like cervical cancer to be a punishment from God and believe that prevalence of cervical cancer is very low among children of God; hence the need for involvement of religious stakeholders in advocacy for integration of cervical cancer screening into routine HIV care.

HIV-infected women with “Much Above Average” perceived risk of developing cervical cancer accepted integration of cervical cancer screening into routine HIV care. This study finding highly correlates with findings from the systematic review conducted in Ethiopia where perceived susceptibility to acquiring cervical cancer (AOR = 3.26; 95% CI : 2.26, 4.26) was significantly associated with cervical cancer screening acceptability among HIV-positive women [35]. However, a study conducted among HIV-infected women reported that perceived risk of acquiring cervical cancer was not significantly associated with acceptability of integration of cervical cancer screening into routine HIV care [8]. Nonetheless, a study conducted in Ghana reported that perceived susceptibility to acquiring cervical cancer was not significantly associated with willingness and acceptability to undergo cervical cancer screening among HIV-infected women [36]. Discrepancies in the influence of perceived risk of cervical cancer to acceptability of integration of cervical cancer screening into routine HIV care could be explained by the gap of lack of awareness that HIV infected women are more at risk of developing cervical cancer compared to their counterparts, HIV negative women.

It was also found out HIV-infected women who had not undergone cervical cancer screening were less likely to accept integration of cervical cancer screening into routine HIV care compared to HIV-infected women who had undergone cervical cancer screening. A study conducted among HIV-infected women at the HIV treatment centre, Nigerian Institute of Medical Research, Lagos, Nigeria reported that ever screened for cervical cancer was not significantly associated with acceptability of integration of cervical cancer screening into routine HIV care [8]. HIV-infected women who have previously undergone cervical cancer screening applaud integration of cervical cancer screening into routine HIV care due to perceived benefits most especially convenience of integrated cervical cancer screening and HIV services. Despite the fact that HIV-infected women who had not undergone cervical cancer screening are highly expected to support integration of cervical cancer screening into routine HIV services, this might not necessarily be true because they could be ashamed of exposing their private parts to health workers whom they occasionally interact with in HIV clinics.

Perceived benefits of the proposed intervention were: convenience to seek for cervical cancer screening, motivation to undergo cervical cancer screening, improved archiving of cervical cancer screening results, confidentiality of HIV patient information, and preference to interact with HIV clinic health workers. In all FGDs, HIV-infected women acknowledged that integration of cervical cancer screening into routine HIV care would grant them an opportunity to receive both cervical cancer screening in addition to HIV services conveniently from the HIV clinic compared to currently practiced referral or client-initiated cervical cancer screening. This study finding is in agreement with findings from other studies which reported that convenience of seeking for integrated HIV and cervical cancer screening services from one point without forgetting an added advantage of one off relief of anxiety was attributed to the integrated strategy compared to stand alone screening services [23, 24]. The intervention was regarded as a time saving strategy attributed to convenience through reducing frequency of health facility visits, necessity of several return journeys, travel time and return transportation costs [23, 24]. HIV-infected women anticipated that implementation of the proposed strategy of delivering cervical cancer screening would reduce disruption and movement from one clinic to another since all cervical cancer screening and HIV related services would be available and received under one roof in a single HIV clinic visit comprehensively.

HIV-infected women in all FGDs reported that integration of cervical cancer screening would motivate HIV infected women to undergo cervical cancer screening services. Furthermore, HIV-infected women in the majority of FGDs alleged that integration of cervical cancer screening into routine HIV care would inspire HIV-infected women, targeted recipients for the proposed intervention, to comply and adhere to recommended annual cervical cancer screening. Researchers, community members, health care providers and policy makers were also optimistic that intervention would improve uptake of initial and annual cervical cancer screening among HIV-infected women [4, 15–24]. Integration of cervical cancer screening into routine HIV services would reduce missed opportunities for HIV-infected women to undergo cervical cancer screening procedure; hence increasing uptake of cervical cancer screening among targeted recipients.

HIV-infected women in the majority of FGDs claimed that the proposed intervention would promote confidentiality of HIV status and any other health related information due to interaction with only health workers and fellow HIV-infected women. This study finding was also reported in a study conducted in Uganda where women reported that the integrated model would promote confidentiality of HIV and any other health related information due to being accessed and confined to limited teams of health care practitioners in HIV clinics [24]. In most instances, HIV-infected women prefer confidentiality of their HIV status through interacting with only health workers in HIV clinics and archiving of cervical cancer screening information in the same file with all other HIV related information; due to fear of being stigmatised.

Shame to expose their privacy to HIV clinic health workers and increased waiting time were the only perceived challenges of integration of cervical cancer screening into routine HIV care among HIV-infected women. HIV-infected women in only “Very Unacceptable and Unacceptable” FGD reported that they would prefer undergoing cervical cancer screening conducted by health workers at the cervical cancer screening unit of Mbarara Regional Referral Hospital as opposed to HIV clinic health workers. Based on a study conducted in Uganda, discomfort and invasion of privacy expressed by HIV-infected women due to genital illnesses, menstrual periods, and fear to expose their private parts was one of the challenges to undergo cervical cancer screening integrated into routine HIV services at Mildmay Uganda [37]. HIV-infected women argued that they would be uncomfortable and ashamed to continue interacting with the same health workers to whom they have exposed their private parts for any other HIV related services.

HIV-infected women in the majority of the FGDs argued that delivering cervical cancer screening comprehensively with other routine HIV care services during scheduled HIV clinic visits would be very time consuming hence increasing waiting time at the HIV clinic. This study finding was consistent with findings from other studies where long waiting time was cited as one of the potential challenges HIV-infected women would have to endure through to undergo cervical cancer screening integrated into comprehensive HIV services [23, 24, 37]. HIV-infected women in some FGDs expressed concern on how the integrated approach of cervical cancer screening and HIV treatment services in a single HIV clinic visit would be delivered to the ever-increasing numbers of recipients within the shortest time possible.

The mixed methods study design using explanatory sequential approach increased rigor and internal validity of the study. Utilisation of systematic sampling method for selection of HIV-infected women to participate in the quantitative phase of the study was an added strength to the conduct of the study. However, the Theoretical Framework of Acceptability adapted to measure acceptability of integration of cervical cancer screening into routine HIV care had not yet been validated among Ugandan populations. HIV-infected women who turned up on unscheduled dates were excluded from participating in the study; increasing potential for information bias. Furthermore, this study was conducted in only one HIV clinic in the entire country due to limited funding; hence affecting generalisability of study findings to all HIV-infected women in Uganda.

## Conclusion

Study findings highlight the need to take advantage of this acceptability to prioritize implementation of integration of cervical cancer screening into routine HIV care. Ministry of Health should prioritise implementation of integration of cervical cancer screening into routine HIV care to increase uptake of cervical cancer screening among HIV-infected women along the continuum of HIV care and treatment services. Health workers delivering HIV care and treatment services should endeavour to conduct intensified health education and awareness about increased risk of developing cervical cancer among HIV-infected women. HIV-infected women should be reassured of reduced waiting time, confidentiality and optimisation of privacy by HIV clinic health workers during provision of integrated HIV care and cervical cancer screening services. Furthermore, a nationally representative study should be conducted to assess acceptability of integration of cervical cancer screening into routine HIV care, associated factors, and perceptions among HIV-infected women enrolled in HIV clinics in Uganda.

## Data Availability

The datasets used and/or analysed during the study are available from the corresponding author on request.
